# Spasm and flexion-relaxation phenomenon response to large lifting load during the performance of a trunk flexion-extension exercise

**DOI:** 10.1186/s12891-017-1869-6

**Published:** 2017-11-29

**Authors:** Yanjun Ma, Xinhai Shan

**Affiliations:** Biomechanics Laboratory, College of Physical Education, Shandong Normal University, 88 Wenhua East Road, Jinan, Shandong 250014 People’s Republic of China

**Keywords:** Low back pain, Lifting load, Trunk flexion-extension, Erector spinae, Muscular intensity

## Abstract

**Background:**

The flexion relaxation phenomenon (FRP) has been widely investigated. Nevertheless, no study has been reported on the FRP as well as spasm response to large lifting load. The aim of this study was to evaluate the effect of large lifting load on the FRP response and spasm during execution of a flexion-extension exercise.

**Methods:**

Twenty-two healthy male university students without low back pain history participated this study. **S**ubjects randomly performed three trials of trunk flexion-extension cycles of 5 s flexion and 5 s extension in each of 4 conditions (three large lifting loads of 15, 20 and 25 kg and one lifting load of 0 kg for comparison). Surface EMG from bilateral erector spinae was recorded during the performance of a trunk anterior flexion-extension exercise. The relaxation phase was determined through the onset of electromyography (EMG) signals. Spasm was evaluated in the relaxation period. The mean normalized electromyography (NEMG) was derived from the raw EMG.

**Results:**

Spasm was observed in more than 45% of the individuals and the intensity of muscle activation was increased by more than 78% in the relaxation phase.

**Conclusions:**

A large lifting load could lead to a high prevalence of spasms as well as a high intensity of muscle activations on erector spinae muscle in the relaxation period, which may be associated with the development of low back disorder during the performance of a flexion-extension exercise.

## Background

Low back pain (LBP) is a serious public health problem in many developed countries [21]. In the United States, for instance, according to a recent report from the Center for Disease Control and Prevention (CDC), back injury in adults is ranked second among the most common reasons that lead to disability [5]. LBP is reported to be responsible for 13% of sick days of all the working population [2], as well as the over 90 billion dollars in annual medical expenses [14, 18]. Despite this, the exact etiology of LBP is still poorly understood [3].

Epidemiologically, trunk bending as well as lifting activities is thought to be a risk factor for the development of LBP [19, 35]. In a previous experimental investigation, Solomonow et al. [31] found that during sustained static lumbar flexion, bilateral spasms were occasionally observed on the erector spinae (ES). In addition, after sustained static lumbar flexion, the relaxation period (from the time of the EMG-off in the flexion phase to the time of the EMG-on in the extension phase) in the FRP on the ES was found to be shorter during the execution of a trunk anterior flexion-extension. Based on the mechanism of a synergistic load sharing between passive tissues and active muscles (e.g., ES) in the FRP [8–10], Solomonow et al. [31] pointed out that the increase of ES muscle activation would indicate the compensation for the decreased ability to resist the tension of stretching passive tissues, which might be closely related to LBD. Recently, an increase of muscle activation in the FRP has also been found after prolonged static compression [28]. The increase of the muscle activation is postulated to be associated with the relative lengthening of ligaments due to the shrinkage of intervertebral discs in parallel with the neural adaptation (synergy or inhabitation) in response to changes in the mechanical properties of the passive tissues, which may be damaged to some degree after prolonged compressive loading [28]. Accordingly, the load on the spine may be a contributory factor to the development of LBD [4, 13, 16, 24].

As a common activity in daily life and sports practice, load lifting increases the external moment in the lumbo-sacral joint [32] as well as the load on passive tissues [7, 12]. However, as it is widely known, no spasm on ES muscle has been reported to occur during the execution of trunk flexion-extension exercises with load lifting. Spasms are random and unpredictable EMG discharge signals in intensity and/or duration time. They might be a strong LBD indicator, representing some type of micro-damage in passive tissues (dorso-lumbar fascia and posterior ligaments) [31, 33].

Previous investigations on loaded trunk flexion-extension exercises have used less than 10 kg of lifting load. However, it was found that the load seemed to have no effect on the FRP [25]. Actually, the authors speculated that the results about the FRP response might be different when the lifting load was larger (>10 kg).

Accordingly, the purpose of the present study was to evaluate the occurrence of spasms and the effect of large lifting load on the FRP response. It was hypothesized that, with a large lifting load, spasms may be observed on bilateral ES muscles during the relaxation phase in the FRP. It was also anticipated that, in the relaxation period, muscle activation intensity would significantly increase in order to compensate for the load increase in passive tissues.

## Methods

### Participant descriptions

Twenty-two male subjects were recruited from the University’s student population to participate in the study after approval by the local ethics committee. Participants read and signed a consent form before participating in the study. Demographic information was collected using a questionnaire to assess prospective participants for eligibility according to the inclusion and exclusion criteria. Their age, weight and height (mean (SD)) were 24(1) years, 71(6.5) kg, 177(5) cm, respectively.

Participants without current complaints of back pain were included in the study. Exclusion criteria included any uncorrectable spine pathology, history of spine surgery, hip conditions that would not allow participants to fully flex and extend their hips comfortably, current back pain, consultation of a physician for back pain in the last year.

### Flexion relaxation measurements

The pre-gelled (Ag-AgCl) disposable surface EMG electrodes were applied at the L3-L4 level over the bilateral ES muscles (about 4-6 cm lateral from the midline). Inter-electrode distance was 2.5 cm, and the electrodes were oriented longitudinally along the muscle. A reference electrode was placed on the left anterior superior iliac crest. The EMG signals were amplified (Biovision, Wehrheim, Germany) × 1000 at a frequency band-pass of 10-500 Hz, 1 μV noise referred-to-input, and CMRR of 120 dB. The Input impedance was 10^9^ kΩ. The resulting signal was sampled at 1000 Hz via a 14-bit data acquisition system and stored for later processing.

### Experimental protocol

The skin was cleansed and gently abraded with alcohol prep pads before attachment of the EMG electrode. The electrodes were placed as described above, and a signal check was conducted to ensure the quality of the EMG signals.

Before trunk flexion-extension, maximum voluntary contraction (MVC) was obtained for the left and right ES muscles for 5 s in one repetition by applying resistance in the Beiring–Sorensen position [6, 20].

**Fig. 1:**
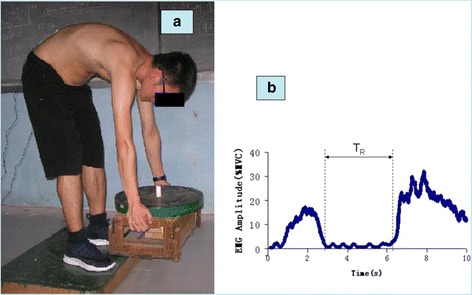
**a**. A subject during flexion phase of a flexion–extension test in 15 kg lifting condition. **b**. The exemplar averaged bilateral EMG amplitude (MVC%) of a participant during flexion–extension performance with 0 kg hand load condition. The parallel vertical dotted lines represent EMG-off (left) and EMG-on (right) timing. T_R_ = the time of relaxation period from EMG-off to EMG-on

The subject was then required to stand on a wood frame with the same height as the customized wooden box, and to perform a trunk flexion-extension while standing, randomly in each of 4 conditions (three large lifting 15, 20 and 25 kg and one lifting load of 0 kg). The box was designed by adjusting the amount of weight to be about 5 kg with dumbbells, which were set through a wooden rod that was fixed inside the box (Fig. 1a). The total amount of lifting load was reached by putting a barbell above the box. For the safety reasons, the barbell was also set through the rod. During the performance of the exercises, all participants were required to put their feet shoulder width apart, keep their knees straight during the test, and contact the toes of feet with their finger nails (in 0 kg) or with the box contacting the floor lightly in full flexion.

Each trial consisted of 10 s total time: 5 s from upright posture to full anterior flexion, and 5 s from full flexion back to upright posture [28].

The timing for each trial was set by a metronome with one beat per second. Each participant performed a full flexion trial, with 2 min rest between each load condition.

### Data analysis

EMG signals were full-wave rectified, then dual-pass filtered through a fourth order Butterworth filter with an effective cutoff frequency of 6 Hz [34] to obtain the mean absolute value (MAV). For MVC, the value was achieved by averaging the MAV in the middle 3 s (eliminating 1 s at the beginning, and 1 s at the end of the total 5 s). For each trial, the MAV was normalized to the MVC value to get EMG amplitude (% MVC) bilaterally. Then, EMG amplitudes from bilateral ES muscles were averaged to represent the bilateral ES muscle activations [17, 27]. A threshold level of 3% of the MVC was used to determine the relaxation period (the beginning and the end of the myoelectric activity) (Fig. 1b) [29].

The NEMG [29] was utilized to represent the intensity of the muscle activation, which was calculated as an averaged EMG (MVC%) in the relaxation phase using the following Equation (Eq.):

1 $$ \mathrm{NEMG}=\frac{1}{N}\sum \limits_{i=1}^N{EMG}_i $$

Where, N denotes the length of the relaxation phase (T_R_ in Fig. 1b). NEMG values were normalized by the 0 kg load condition (Table 1).

**Table 1:** Statistical results of Spasms and NEMG in relaxation period

Lifting Load (kg)	Spasms		NEMG(%MVC) (Mean(SD))	
	Case	prevalence	Absolute (%MVC)	Normalized
0	0	0	1.05(0.21)	1.00(0.00)
15	10^a**^	45%^a**^	1.87(0.33) ^a**^	1.78(0.09) ^a**^
20	15^a**b*^	68%^a**b*^	2.13(0.32) ^a**^	2.03(0.10) ^a**b*^
25	18^a**b**c*^	82% ^a**b**c*^	2.37(0.40) ^a**b**^	2.26(0.12) ^a**b**c*^

** *P* < 0.01;*P < 0.05 (a = vs. 0 kg; b = vs. 15 kg; c = vs. 20 kg). (NEMG = mean normalized EMG)

**Fig. 2:**
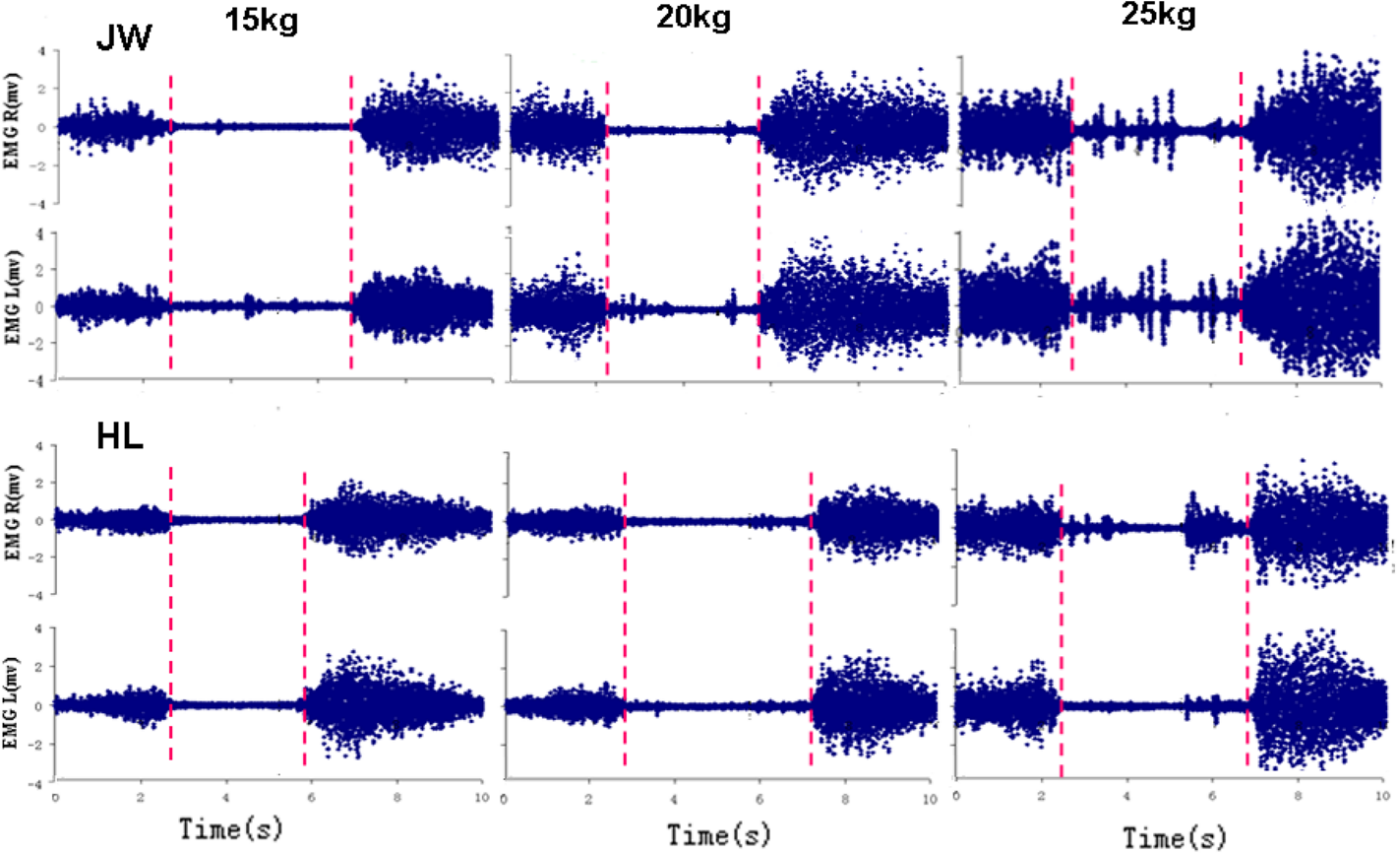
Two samples (JW and HL) of bilateral EMG recordings from ES during flexion-extension performance with large lifting load condition, respectively. During relaxation period in 15 kg condition, subject JW shows some spasms in a form of low amplitude with sustained discharge mainly on left ES while HL having no spasms. In 20 kg condition, JW shows some spasms in form of sporadic potential discharge mainly on left ES while HL having a little spasm. In 25 kg, both JW and HL shows the spasms obviously triggered with sporadic large amplitude action potential discharge bilaterally. The vertical parallel dotted lines represent EMG-off (left red) and EMG-on (right red) timing

The evaluation of spasms during the relaxation period is performed in two steps. The first step was a qualitative analysis. Spasm-like signals were preliminarily separated from the raw signals of all trials if there were random and unpredictable EMG signals in intensity and/or duration time (Fig. 2). The second step was a quantitative analysis. A threshold, twice the NEMG signal with the 0 kg load condition during the relaxation period was utilized to ultimately evaluate the spasms.

The SPSS software version 15.0 (SPSS, Inc., Chicago,IL, USA) was used for all statistical analysis. To test the effect of a large load, a Chi-Square test was performed on both the case and prevalence of spasms between groups. Additionally, repeated measures analysis of variance (ANOVA) test was used on both the absolute and normalized NEMG. A post-hoc test with a Bonferroni adjustment was also used to compare differences in the NEMG between groups. Significance was set at *p* ≤ 0.05 for all measurements.

## Results

A significant effect of large load lifting (*p* < 0.05) was found both on the case and the prevalence of spasms. Among the total 22 participants, there were 10, 15 and 18 spasm cases in the load conditions of 15, 20 and 25 kg with a prevalence of 45%, 68% and 82%, respectively (Table 1).

In the relaxation period, significant effect of large load lifting (*p* < 0.05) was also found both on the absolute and normalized NEMG. With the increase of the lifting load, the normalized NEMG exhibited a significant increase (*p* < 0.05). The normalized NEMG in large lifting experienced an increase of 78%–126% compared with that in 0 kg load lifting.

## Discussion

The main results of this study showed that, during anterior trunk flexion-extension with a large lifting load, spasms occurred with a high prevalence, and the normalized NEMG showed a significant increase in the relaxation period.

Unlike the finding by Sarti et al.[25], which indicated that a light load (<10 kg) has no effect on FRP, a significant effect of a large load on the normalized NEMG signal was detected in the relaxation period. Compared with the 0 kg load, the NEMG signal increased 78% with the 15 kg whereas the increased signal intensity reached 126% with the 25 kg. It was suggested that the NEMG signal could represent the intensity of the muscle activation during the relaxation phase [29]. They suggested that the higher NEMG in the ES muscle during the relaxation phase might be linked to fatigue during sustained trunk flexion. However, it is obvious that fatigue was not the case in the study since the participant had enough rest time between trials and the performance of the exercise in one trial lasted only 10 s. Thus, it was more likely to be a sustained muscle activity, which was commonly detected in back pain patients with ES muscle activity ([10]; Floyd and Silver, 1955). Therefore, muscle activation related to the large load lifting during the relaxation period may indicate a compensation for passive tissues [15, 26], which is closely related to LBD [4, 13, 31].

When lifting a large load, spasms were observed in more than 45% of participants with the 15 kg load, or even reached to 82% with the 25 kg load. Also, the larger the lifting load was, the higher the prevalence of spasm was. Spasm is usually associated with some type of micro-damage developing in the passive viscoelastic tissues of the lumbar spine [31, 33]. The damage of ligamentous tissue could trigger spasms in the associated muscles [22], which is manifested by LBD, a clinical symptom in patients [1, 11, 30].

There were some limitations that may affect the generalization of the results of this study. First, there were no female participants who are thought to show a different response under the same load lifting conditions [23, 31]. Second, the load was the same for every participant. The response to a large load would probably be different if the load, relative to body mass, was lifted during the performance of a trunk flexion-extension exercise as Plamondon et al. [23] suggested. Third, the weight variation of the large load lifting in this study was only from 15 to 25 kg. For safety reasons, a load >25 kg was not selected. However, according to the tendency of spasm and muscle activation in NEMG signical in the study, it could be considered that the higher prevalence of spasms and higher muscle activation intensity could occur when the lifting load was >25 kg. Fourth, this study involved only EMG. In future studies, investigating the kinetic and kinematical variables, together with EMG, may be helpful for us to understand the mechanism of load transfer in the lumbar spine under a large load condition similar to the investigation by Howarth and Mastragostino [13].

## Conclusions

The general conclusion drawn from the results of this study is that during the performance of a trunk flexion-extension exercise with a large lifting load, the muscle activation intensity of the bilateral ES significantly increased to compensate for passive tissues during the relaxation period. In addition, spasms on bilateral ES muscle were observed in more than 45% participants. The significant increase tendency in muscle activation together with a high prevalence of spasms indicates that a low back disorder may develop during the performance of a flexion-extension exercise with a large lifting load.

## Abbreviations

CDC: Center for disease control and prevention
EMG: Electromyography
ES: Erector spinae
FRP: Flexion relaxation phenomenon
LBD: Low back disorder
LBP: Low back pain
MAV: Mean absolute value
MVC: Maximal voluntary contractions
NEMG: Mean normalized EMG
